# Environmental DNA sheds new insight on molecular adaptation of foraminifera to temperature from laboratory‐controlled culture experiment

**DOI:** 10.1002/ece3.70243

**Published:** 2024-10-10

**Authors:** Haotian Li, Yanli Lei, Wenlong Fa, Tianzhen Wu, Tiegang Li

**Affiliations:** ^1^ Laboratory of Marine Organism Taxonomy and Phylogeny, Qingdao Key Laboratory of Marine Biodiversity and Conservation Institute of Oceanology, Chinese Academy of Sciences Qingdao China; ^2^ Laboratory for Marine Biology and Biotechnology Qingdao Marine Science and Technology Center Qingdao China; ^3^ Southern Marine Science and Engineering Guangdong Laboratory (Zhuhai) Zhuhai China; ^4^ University of Chinese Academy of Sciences Beijing China; ^5^ Key Laboratory of Marine Sedimentology and Environmental Geology First Institute of Oceanography, MNR Qingdao China

**Keywords:** benthic foraminifera, laboratory‐controlled culture, molecular adaptation, paleotemperature reconstruction

## Abstract

Foraminifera is the most important temperature proxy of the ocean on long time scales. However, the absence of temperature‐controlled experiments at different water depths hinders the advancement of paleotemperature reconstruction with foraminifera from the continental shelf. For the first time, this study investigated the response of benthic foraminifera to temperature change using microcosm culture and metabarcoding. Foraminiferal communities from three continental stations at varying water depths (6.0, 9.2, and 26.0 m) were cultured under five temperature gradients (6, 12, 18, 24, and 30°C), with each treatment performed in triplicate. The foraminifera were fed with microalgae every 4 days, and the filtered seawater (through 0.22 μm pores), acting as a medium, was changed accordingly. The experiment lasted for 80 days, and 47 DNA samples were obtained and analyzed, including three in situ samples. The results showed that foraminifera adjusted its growth rate within the low‐temperature range and adopted an r‐strategy to cope with high‐temperature stress. In addition, the foraminifera from deeper water stations exhibited a pronounced vulnerability to diminishing read counts. The read counts, operational taxonomic units (OTU) counts and Margalef index of foraminifera and the read counts of Rotaliida exhibited a remarkably positive correlation with temperature. The recommended relationships were described as read counts = 1314.75*T + 44754.51; OTU counts = 1.13*T + 44.26; Margalef index =1.13*T + 44.26. This study established the first quantitative relationship between temperature and foraminifera molecular parameters that holds significant implications for long‐time paleotemperature calibration in climate change.

## Graphical abstract

For the first time, the adaptability of foraminifera to temperature changes was revealed from the view of molecular biology, and we established the quantitative relationships between foraminiferal molecular parameters and temperature.

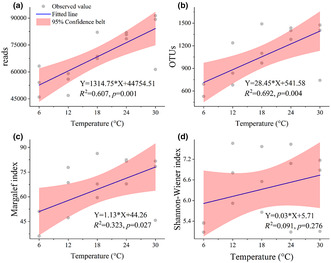

## 1 INTRODUCTION

Establishing quantitative relationships between benthic foraminifera and environmental factors has become an important method in paleotemperature reconstruction (Rosenthal et al., 1997). Many studies have explored the relationship between foraminifera and sea temperature, with a particular focus on species (Barras et al., 2010; Evans et al., 2015). They studied the oxygen isotopic composition of *Bulimina marginata* under controlled experimental temperature range (4–19°C) (Barras et al., 2010). Another study presented Mg/Ca–temperature laboratory calibrations of *Globigerinoides ruber* (Evans et al., 2015). These studies focused on the shell isotope of foraminiferal individuals. In 2011, Goldstein and Alve demonstrated that benthic foraminiferal communities (*Ammonia tepida*, *Haynesina germanica*, *Elphidium excavatum*, etc.) can reproduce normally at temperatures 12°C. Li et al. (2019) and Dong et al. (2019b) described the response (abundance, diversity, etc.) of intertidal foraminiferal communities to different temperatures. In shallow waters, the calcareous foraminifera can even settle 43 million tons of carbonate per year (Langer, 2008), which accounts for approximately 1% of global carbonate sedimentation (Langer et al., 1997). Thus, Rotaliida (calcareous foraminifera) might be a potential carbon sink. Furthermore, carbon dioxide emission from anthropogenic activity is an important factor of acceleration in global temperature following the Industrial Revolution, which is of great human concern. The increased carbonate sedimentation of calcareous foraminifera will offset the impact of anthropogenic emissions on climate change. Therefore, the relationship between foraminifera with temperature must be considered for palaeotemperature reconstruction in long‐term climate change. However, collecting benthic foraminifera from continental shelf areas is challenging due to several factors, such as water depth and limitations of sampling tools. In addition, relatively few successful cases (Dong et al., 2019a) of culturing benthic foraminifera from these areas in laboratory settings have been reported. As a consequence, systematic studies on the response of foraminifera to temperature changes in continental shelf sediments are lacking (Antão et al., 2020).

Environmental DNA (eDNA) is genetic material obtained from environmental samples such as soil, sediment and water (Thomsen & Willerslev, 2015). It includes a wide range of organisms, including microbes, plants, vertebrates, protozoa, and others (Pedersen et al., 2015). Next‐generation sequencing, also known as high‐throughput sequencing, provides a new approach to investigating the abundance and diversity of foraminifera (He et al., 2019; Lecroq et al., 2011; Lejzerowicz et al., 2013), particularly in small or damaged individuals of foraminifera (Pawlowski & Lecroq, 2010). This technology has been applied for monitoring environmental changes in various fields, such as aquaculture (Pawlowski et al., 2014; Pochon et al., 2015), marine pollution (Frontalini et al., 2018), oil drilling (Laroche et al., 2016), and sea level change (Moss et al., 2016), and has a wide application prospect in the study of ancient DNA. By analyzing the DNA of foraminiferal fossils, researchers can reconstruct past environmental conditions and provide insights into the evolution of marine ecosystems (Pawłowska et al., 2014, 2016). However, the quantitative relationship between foraminiferal molecular parameters (Margalef, Shannon–Wiener index, etc.) and environmental factors has not been reported, thus limiting the application of foraminiferal molecular research in paleotemperature calibration. Therefore, further studies are crucial to address this issue.

We conducted laboratory‐controlled culture experiments to explore the molecular response of benthic foraminiferal communities from the continental shelf to temperature change. We used the benthic foraminifera community from Jiaozhou Bay as an example. Our goal was to determine (1) the relationship between the molecular parameters of foraminifera and temperature and (2) identify which parameter can be used as a proxy for temperature. Through our experiments, we revealed the molecular adaptability of foraminifera to temperature changes and established the quantitative relationships between their molecular parameters and temperature for the first time. The relationships were described as follows: foraminiferal read counts =1314.75*T + 44754.51; OTU counts =1.13*T + 44.26; Margalef index =1.13*T + 44.26. Our findings provide a reference for studying the molecular adaptation of carbonate organisms to global warming and applying the relationships between foraminiferal molecular parameters and temperature to paleotemperature reconstruction on long time scales.

## 2 MATERIALS AND METHODS

### 2.1 Sample collection

From August 16 to 17, 2018, sediment samples were collected from Jiaozhou Bay (Figure S1a,b) using a 0.1 m^2^ box sampler aboard the scientific research ship “Innovation.” The samples (top 1 cm sediments) contained living foraminifera were collected from the shallow water shelf using a spoon. Sediment temperature was measured using a spirit thermometer (precision: 1°C). The recorded mean temperature of the three stations was 24.2°C (A5: 24.1°C; C4: 24.3°C; D6: 24.0°C). Salinity was measured with a portable refractometer (precision: 1 psu). The measured salinity value of the three stations on average was determined to be 30.2 psu (A5, C4, D6: 30.2 psu). Stations A5, C4, and D6 were chosen as representative stations based on the geographical location, with A5 being located in the inner waters of Jiaozhou Bay at a depth of 9.2 m, C4 at the mouth of the bay at a depth of 6.0 m, and D6 in the outer waters of Jiaozhou Bay at a depth of 26.0 m. The water depth data were obtained from CTD onboard the “Innovation.”

### 2.2 Experimental design

After being collected, the sediments containing living foraminifera were transported to the laboratory in 500 mL plastic bottles filled with seawater from the sampling site. In the laboratory, the sediments from the same stations were mixed carefully and thoroughly using a spoon in a clean plastic wash basin (40 cm diameter, 13 cm height). Afterward, 1‐cm‐thick portions of sediment were scooped out and randomly placed in a series of columnar glass cylinders (9 cm diameter, 5 cm thickness), along with 3 cc of filtered (through a 0.22‐μm‐pore filter) seawater. The glass cylinders were sealed with fitting plastic lids to reduce evaporation during the experiment (Goldstein & Alve, 2011). The foraminifera were fed with a mixture of *Isochrysis galbana* 8701 and *Phaeodactylum tricornutum* (Bernhard et al., 2004; Diz et al., 2012), which were precultured in an f/2 medium. A 2 days preincubation under formal experimental conditions was conducted to minimize biological stress.

The experiment included five temperature treatments (6°C, 12°C, 18°C, 24°C, and 30°C) and three stations (A5, C4, and D6), with each treatment performed in triplicate (Figure S1c). Forty‐five samples (5 temperatures × 3 stations × 3 replicates) were cultured. The experiment was conducted in temperature‐controlled incubators (Wuhan Ruihua Instrument & Equipment Co., Ltd.; Ruihua; HP250G‐D; volume 250 L; precision ≤ ± 1°C) under 2000 lux, with a 12 h light/dark cycle. The cycle conditions were designed to emulate the variations in light exposure that typically occur in natural environments. The foraminifera were fed with 1 mL of concentrated, freeze‐dried *I. galbana* 8701 and *P. tricornutum*, and the 0.22‐μm‐pore filtered seawater was changed every 4 days to maintain stable salinity. The experiment lasted for 80 days, and three replicates of sediments from each station were preserved as in situ samples before the experiment (T0). Finally, 48 samples were obtained.

### 2.3 Sample treatment

#### 2.3.1 Total genomic DNA extraction

After the culture experiment, total genomic DNA in the sediment samples was extracted using the DNeasy Power Soil DNA Extraction Kit (Qiagen, Germany). Three replicates of DNA samples with one blank control (without sediment sample) were extracted from each sediment sample. About 0.25 g of sediment from each replicate was weighed. All steps were performed in accordance with the manufacturer's instructions.

#### 2.3.2 PCR amplification and sequencing

The foraminiferal SSU rDNA fragment was amplified using the foraminifera‐specific primer pair s14F1 (5′ AAGGGCACCACAAGAACGC 3′) and s17 (5′ CGGTCACGTTCGTTGC 3′) (Lejzerowicz et al., 2013). The PCRs were performed as follows: predenaturation at 94°C for 90 s, denaturation at 94°C for 30 s, annealing at 55°C for 60 s, and extension at 72°C for 45 s. This process lasted for 35 cycles, with a final elongation at 72°C for 3 min. The reaction system included 12.5 μL of 2× High‐Fidelity Master Mix, 0.5 μL of 10 μM primer, 2 μL of DNA template, and 9.5 μL of ddH2O. For the detection of cross‐contamination, the PCR without DNA template was used as a negative control. The PCR products were detected by electrophoresis using 1% agarose gel, and the target fragment was about 300 bp. All blank and negative controls remained negative throughout the experiments. Sequence libraries were generated using the TruSeq® DNA PCR‐Free Sample Preparation Kit from Illumina (NEB, USA) in accordance with the manufacturer's instructions. The library quality was detected using a Qubit 2.0 Fluorometer and Agilent BioAnalyzer 2100 system. The library was sequenced on an Illumina NovaSeq platform, and 250 bp paired‐end sequences were generated. Sequencing was performed by Beijing Novogene Biotech Co., Ltd.

#### 2.3.3 Quality control and data processing

To ensure accuracy and reliability, quality control was carried out on the raw sequencing data due to interference. First, the data were separated into samples based on the barcode sequence and primer sequence, and the barcode and primer sequence were then removed. The raw paired‐end sequences of each sample were joined using Flash (Version 1.2.7) (Magoč & Salzberg, 2011), and the joined sequences were called raw sequences. High‐quality filtering, chimeras, and low‐quality removing were carried out on the raw sequences to obtain high‐quality, effective data (effective sequences) based on QIIME (Version 1.9.1) (Caporaso et al., 2010) and Usearch (Version 11.0.667) (Edgar, 2013). OTUs (Operational Taxonomic Unit) were generated via an unoise3 pipeline (Edgar, 2016). An OTU table was generated using Usearch (Decelle et al., 2014; Tragin et al., 2018). A unique sequence was kept in each of the OTUs as a representative sequence. All the representative sequences of OTUs were assigned to the PR^2^ reference database (Protist Ribosomal Reference database; Version 4.12.0) (Guillou et al., 2013) using BLAST (Version 2.7.1) (Altschul et al., 1990) to obtain the classification information of the OTUs. The OTUs were assigned to specific foraminiferal species. Those OTUs that were not assigned to foraminifera were collectively labeled as “Other.” The sequences produced by sequencing become read. The number of sequence (read) for each OTU assigned to a foraminiferal species was considered the read count for that species. These read counts can be used to represent the relative abundance of the corresponding foraminiferal species. It is important to note that some factors like the variation in rDNA copy number could affect the interpretation of the read counts for the foraminiferal species (Milivojević et al., 2021).

### 2.4 Statistical analysis

The sequencing data were normalized on the basis of the smallest amount of data in the sample. The analysis of alpha diversity indices, such as Shannon–Wiener and Margalef, was performed based on OTU table data. The data were standardized by resampling based on the minimum number of OTUs across the samples. This step ensured the data were comparable across samples. Then, these diversity indices were calculated using the “alpha_diversity.py” command in the QIIME software (Caporaso et al., 2010). Plotting analysis was performed with R (version 4.0.0). To explore the relationship between foraminiferal community, Spearman correlation between foraminiferal molecular parameter (read and OTU counts, Shannon–Wiener and Margalef index, etc.) and temperature was analyzed using Statistical Package for the Social Sciences (SPSS, version 22.0). Regression analysis was conducted in Origin (version 2021) to model the quantitative relationship between the foraminiferal molecular parameter and temperature.

## 3 RESULTS

### 3.1 Data review

We obtained 4,031,935 raw sequences from the raw data and 3,885,880 clean sequences and 3,871,615 effective sequences after removing low‐quality sequences and strict quality control (Table S1). Finally, 3867 OTUs were reserved in the OTU table (Table 1; Table S2). As shown in Figure S2, the rarefaction curves rapidly increased when the number of DNA sequences obtained from each sample were less than 10,000. However, the curves increased slowly when the read counts exceeded 30,000. This finding suggested that using additional data could only produce a few new species.

**TABLE 1:** The foraminiferal parameters with SDs in temperature treatments (6, 12, 18, 24, and 30°C) at different stations (A5: Water depth of 9.2 m, C4: Water depth of 6.0 m, and D6: Water depth of 26.0 m).

Station	Treatment	Read counts	OTU counts	Margalef index	Shannon–Wiener index
A5	in situ	82679.00 ± 4221.43	1055.00 ± 65.05	67.85 ± 6.26	7.36 ± 0.43
	6°C	45967.33 ± 23130.71	691.00 ± 694.77	51.10 ± 52.12	5.35 ± 3.04
	12°C	55934.67 ± 9563.19	678.33 ± 154.72	47.27 ± 12.28	5.92 ± 0.74
	18°C	68625.33 ± 15999.99	1100.00 ± 623.88	67.79 ± 37.82	6.55 ± 2.01
	24°C	78451.00 ± 4156.82	1402.67 ± 94.48	81.64 ± 6.12	7.63 ± 0.31
	30°C	91330.00 ± 7227.76	1474.67 ± 53.00	81.71 ± 5.79	6.88 ± 0.23
C4	in situ	78826.00 ± 3860.64	1125.67 ± 75.94	69.36 ± 2.39	7.28 ± 0.11
	6°C	54067.67 ± 8251.13	527.67 ± 43.84	36.08 ± 0.72	5.08 ± 0.09
	12°C	20725.33 ± 2506.93	454.00 ± 66.73	39.18 ± 6.34	4.43 ± 0.62
	18°C	67473.67 ± 6987.73	970.67 ± 489.02	59.52 ± 28.82	5.66 ± 2.54
	24°C	80778.00 ± 9477.94	1436.33 ± 121.46	82.48 ± 8.72	7.07 ± 0.91
	30°C	89259.67 ± 4627.65	1406.00 ± 105.06	78.43 ± 10.42	7.17 ± 0.40
D6	in situ	71598.33 ± 10242.67	1101.33 ± 42.00	67.17 ± 0.49	5.88 ± 0.54
	6°C	63228.67 ± 12435.93	528.00 ± 58.64	36.19 ± 6.22	5.34 ± 0.53
	12°C	27426.00 ± 27462.11	658.00 ± 502.78	51.39 ± 22.92	5.18 ± 1.43
	18°C	82084.67 ± 4276.56	1490.67 ± 50.85	86.34 ± 4.49	7.57 ± 0.12
	24°C	81831.67 ± 2040.32	1284.67 ± 115.22	67.83 ± 7.99	5.08 ± 0.82
	30°C	61381.33 ± 34696.25	741.00 ± 428.86	45.74 ± 21.14	5.10 ± 1.08

*Note*: Each treatment was performed in triplicate.

### 3.2 Relationship revealed with community parameters

**FIGURE 1:**
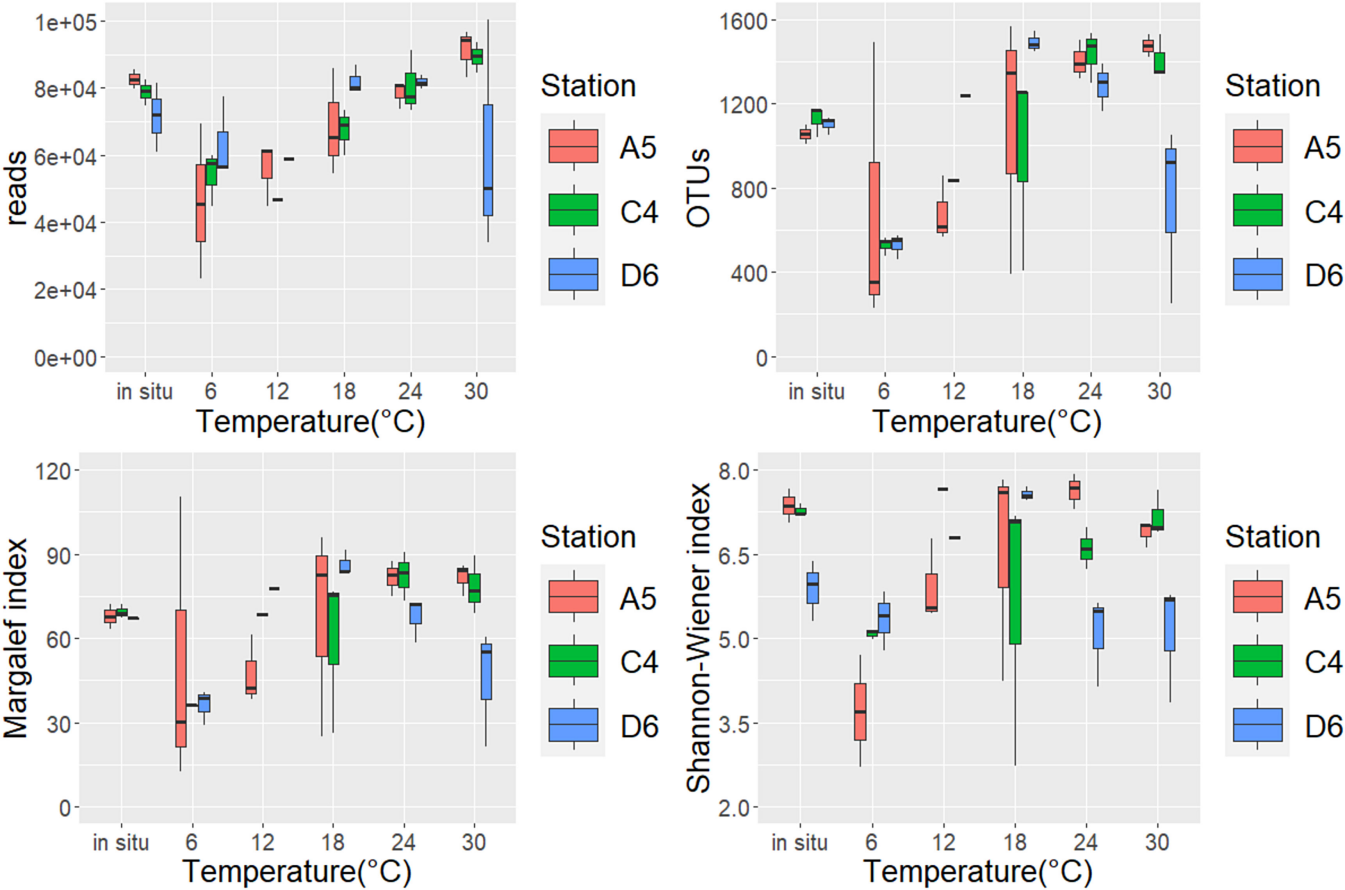
The read counts, OTU counts, Margalef index, and Shannon–Wiener index of living foraminifera plotted against temperature (6, 12, 18, 24, and 30°C) at different stations. A5: Water depth of 9.2 m; C4: Water depth of 6.0 m; D6: Water depth of 26.0 m.

**FIGURE 2:**
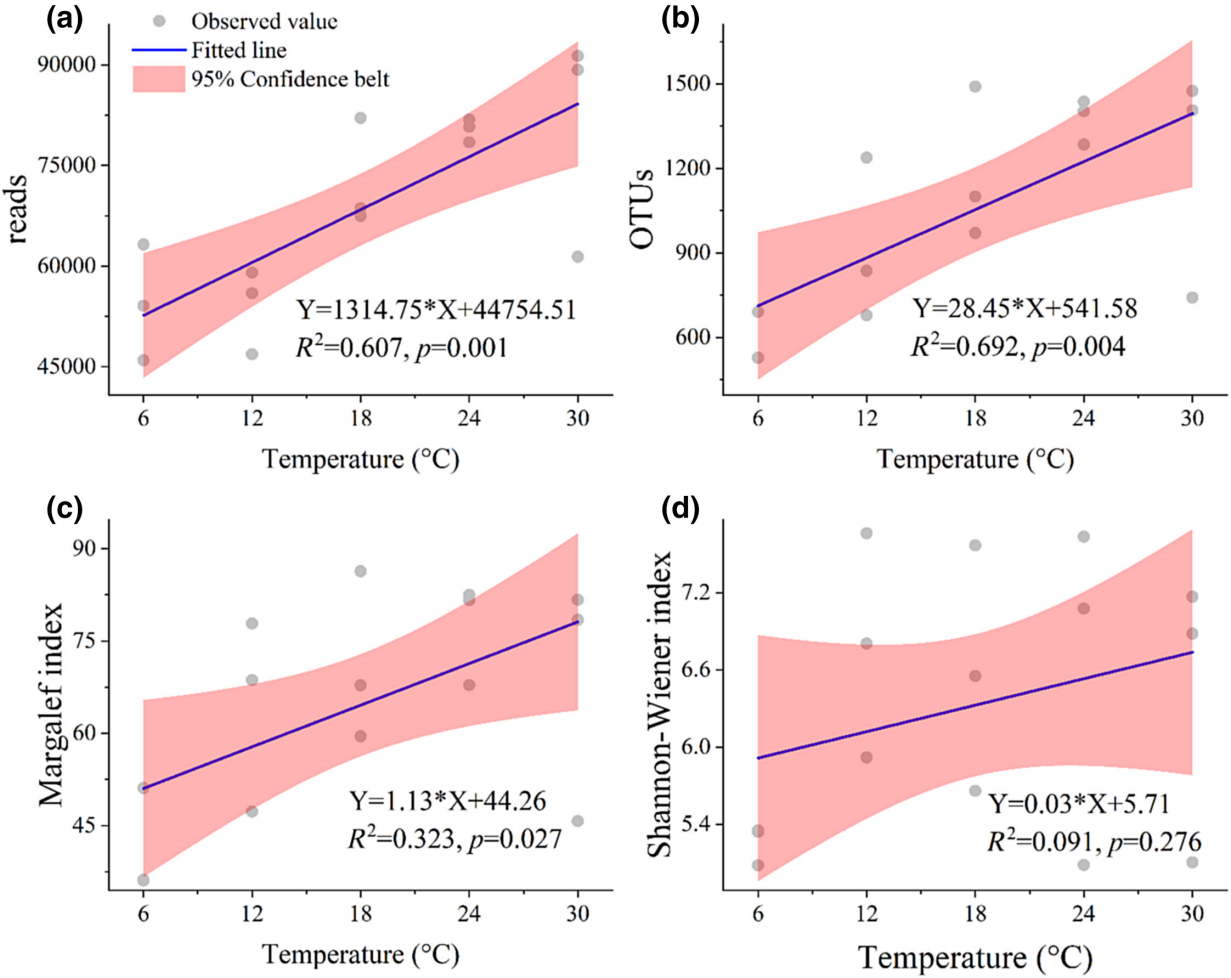
The fitted line of foraminiferal parameters (read counts, OTU counts, Margalef index, and Shannon–Wiener index) plotted against temperature (6, 12, 18, 24, and 30°C).

In this study, the foraminiferal read counts increased with the temperature at different stations (Figure 1). The values ranged from 26,939.00 to 99,958.33 (Table 1) and showed a significantly positive correlation with temperature (Table 2). Regression analysis revealed a good fit between foraminiferal read counts and temperature, with the equation Y = 1314.75*X + 44754.51 (*R* ^2^ = .607, *p* = .001) (Figure 2). The trends of OTU counts and the Margalef index were similar to that of foraminiferal read counts, and correlation analysis confirmed their positive correlation with temperature (Table 2). The equation Y = 28.45*X + 541.58 (*R* ^2^ = .692, *p* = .004) exhibited a good fitting for OTU counts and temperature. Moreover, a significantly fitted line was found between the Margalef index and temperature (Y = 1.13*X + 44.26; Figure 2). However, the trend of the Shannon–Wiener index was different from that of the parameters mentioned above; that is, it initially increased but subsequently decreased when temperature increased and showed no significant correlation with temperature (Table 2). Thus, the relationship between read counts, Margalef index of foraminifera and temperature could be good proxies.

**TABLE 2:** Correlations (Spearman's *r* values) between the foraminiferal parameters (read counts, OTU counts, Margalef index, and Shannon–Wiener index) and temperature.

	Read counts	OTU counts	Margalef index	Shannon–Wiener index
*r*	**.764**	**.698**	**.535**	.284
*p*	.001**	.004**	.040*	.306

*Note*: The *α*‐level is .05. *p*‐values beneath .05 and .01 are considered statistically significant and marked bold and by a single asterisk and double asterisks, respectively.

### 3.3 Relationship revealed with community composition

**FIGURE 3:**
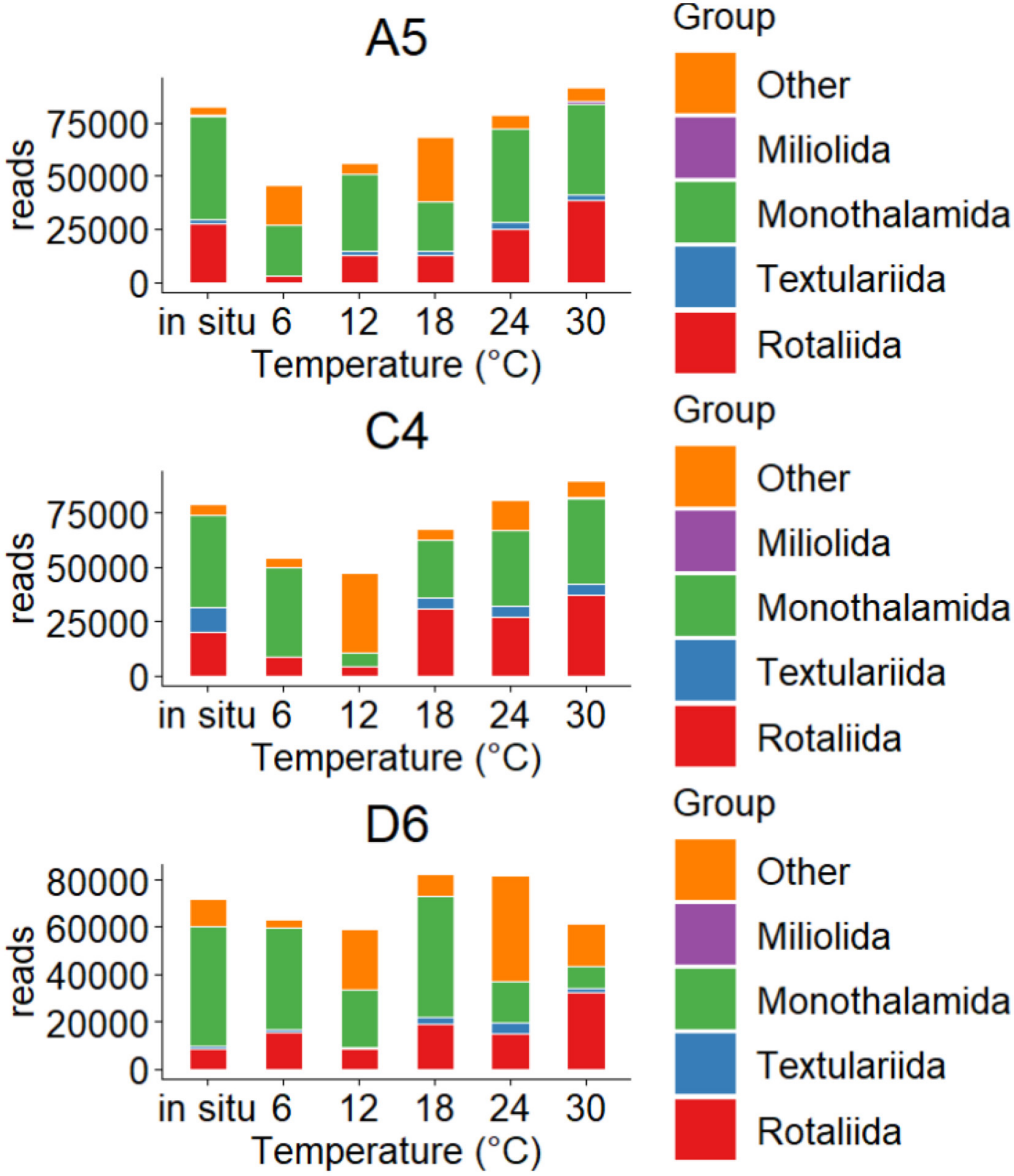
The read counts of four living foraminiferal groups (Rotaliida, Textulariida, Monothalamida, and Miliolida) under different temperatures (6, 12, 18, 24, and 30°C) at station A5 (water depth of 9.2 m), C4 (water depth of 6.0 m), and D6 (water depth of 26.0 m).

The reserved OTUs were annotated using BLAST. A total of 118 foraminiferal species (File  S1) were assigned to PR2 reference database with an identity >90.75% on average. The highest ratio belonged to Monothalamida (45.74% on average), followed by Rotaliida (26.82%). A small number of sequences were annotated to Textulariida, and the ratio of Miliolida was less than 0.2% on average (Figure 3). The read counts of Rotaliida at different stations showed an increasing trend with the culture temperature. The least number of reads was obtained at 6°C, and the most (38,615.00) were obtained at 30°C (Table 3; Table S3). Correlation analysis confirmed a significantly positive correlation between the read counts of Rotaliida with temperature (Table 4). For other shell types, the read counts showed an irregular trend with changing temperature and had no significant correlation with temperature (Table 4).

**TABLE 3:** The read counts attributed to foraminiferal shell groups with SDs in temperature treatments (6, 12, 18, 24, and 30°C) at different stations (A5: Water depth of 9.2 m, C4: Water depth of 6.0 m, and D6: Water depth of 26.0 m).

Station	Treatment	Rotaliida	Textulariida	Monothalamida	Miliolida
A5	in situ	27808.00 ± 4911.56	1887.50 ± 85.56	48634.50 ± 9044.60	215.00 ± 16.97
	6°C	2927.67 ± 1885.62	134.00 ± 57.89	23773.67 ± 17244.57	9.33 ± 6.66
	12°C	12714.67 ± 5639.44	1846.67 ± 1774.81	36033.67 ± 14759.72	73.33 ± 24.58
	18°C	12800.67 ± 10708.07	1840.00 ± 1824.52	23016.67 ± 18487.43	68.33 ± 59.20
	24°C	24976.33 ± 3309.69	2963.33 ± 747.88	43959.67 ± 5650.10	78.33 ± 4.62
	30°C	38615.00 ± 5232.06	2251.67 ± 689.79	42994.00 ± 3420.45	1098.67 ± 840.37
C4	in situ	19658.33 ± 4451.15	11750.67 ± 8225.31	42173.67 ± 5849.45	187.67 ± 205.56
	6°C	8364.67 ± 1434.68	286.33 ± 34.65	41097.00 ± 6101.55	29.00 ± 11.27
	12°C	2578.67 ± 845.00	158.33 ± 113.81	4153.00 ± 1266.81	6.00 ± 4.00
	18°C	30950.00 ± 4359.54	4836.67 ± 5296.45	26658.67 ± 7924.48	92.67 ± 74.97
	24°C	26617.33 ± 11419.62	5676.00 ± 2039.68	34290.00 ± 12901.47	125.00 ± 16.52
	30°C	37163.33 ± 4596.63	5163.33 ± 2336.74	39096.67 ± 5602.04	311.67 ± 146.65
D6	in situ	8391.00 ± 2914.76	1435.00 ± 187.16	50288.33 ± 10135.94	154.00 ± 3.61
	6°C	15299.00 ± 3566.71	1157.67 ± 955.31	43139.33 ± 9206.33	107.00 ± 117.80
	12°C	3333.67 ± 4548.31	196.67 ± 270.57	10415.33 ± 12263.14	42.33 ± 58.77
	18°C	19043.67 ± 3135.21	2571.33 ± 209.55	51299.33 ± 5488.79	138.33 ± 48.95
	24°C	14640.00 ± 3079.66	4760.33 ± 669.87	17287.67 ± 5012.08	70.00 ± 13.86
	30°C	32245.00 ± 25333.20	1554.67 ± 1718.02	9821.33 ± 7372.39	21.33 ± 4.93

*Note*: Each treatment was performed in triplicate.

**TABLE 4:** Correlations (Spearman's *r* values) between the read counts of (Rotaliida, Textulariida, Monothalamida, and Miliolida) and temperature.

	Rotaliida	Textulariida	Monothalamida	Miliolida
Read counts	0.687 (0.005)**	−0.011 (0.969)	0.426 (0.114)	−0.049 (0.862)

*Note*: The *α*‐level is .05. *p*‐values (in the parentheses) beneath .05 and 0.01 are considered statistically significant and marked bold and by a single asterisk and double asterisks, respectively.

## 4 DISCUSSION

### 4.1 Adaptation to temperature changes

The current trend of global warming has attracted significant attention from the scientific community (Perry et al., 2005; Thomas et al., 2004) due to its expected impact on the primary productivity of the ocean. This impact will affect the entire food web, flow of energy, and circulation of matter (Goes et al., 2005), and this effect is particularly pronounced in shallow continental shelf areas (Kwiatkowski et al., 2017).

In this experiment, temperature change showed a significant and positive effect on the read counts of benthic foraminiferal community. When the temperature continued to rise, the read counts of foraminifera increased significantly from 26,939.00 to 99,958.33 (Table 1). However, these results were different from the synchronous morphological data, which suggested that foraminiferal abundance first increased but then decreased with the increasing temperature (Li et al., 2023). In this morphological study, foraminiferal abundance rose from 6°C to 18°C but declined thereafter between 18°C and 30°C, suggesting an ecological inflection point for continental shelf foraminifera at 18°C. In the same experiment, it appeared that morphological data (Li et al., 2023) and eDNA data (this study) revealed disparate response patterns of foraminifera to temperature fluctuations. Previous morphological studies have also found that foraminiferal abundance decreases at high temperatures (>18°C) (Li et al., 2019). It should be noted that the number of eDNA reads does not exactly correspond to the absolute abundance of organisms in the environment, and it may be affected by other factors, such as (1) internal factors of organisms (e.g., copy number of rDNA) (Milivojević et al., 2021), environmental conditions (e.g., the degradation of DNA) (Barnes et al., 2014), and experimental bias (Gardes & Bruns, 1993). Although these factors may introduce some biases, the eDNA read counts can still reflect the relative abundance of different species in the environment (Lacoursière‐Roussel et al., 2016). More importantly, in the face of environmental change (temperature change in this study), the trend of the read counts of eDNA change is consistent with the trend of biological abundance or quantity change in the environment (Yates et al., 2019). We linked the read counts of eDNA with the relative abundance of foraminifera. During low‐temperature range, the read counts (this study) and abundance (synchronous morphological study, Li et al., 2023) both showed positive correlation with temperature. Lombard et al. (2009) found that temperature has a positive effect on foraminiferal growth rate, which has been confirmed in other studies (Lei et al., 2019). Therefore, the low values of foraminiferal abundance (synchronous morphological study, Li et al., 2023) and read counts at low temperatures (this study) are possibly due to a slow growth rate, which increases when the temperature gradually increases. However, foraminifera may face environmental stress and decreasing abundance at high temperatures (Titelboim et al., 2016). In the late Maastrichtian, Keller and Abramovich (2009) found that foraminifera exhibit the “Lilliput effect” in response to environmental stresses, such as greenhouse warming and mesotrophic or restricted basins. Facing these stresses, foraminiferal individuals may opt to shrink in size and produce additional offspring using the r‐strategy (Keller, 2003; Keller & Abramovich, 2009). The small individuals (<150 μm) could be ignored in some morphological studies (Lei et al., 2017; Li et al., 2020) but can be detected by sequencing (Lejzerowicz et al., 2014; Pawlowski et al., 2011). The consistency between the morphological (Li et al., 2023) and molecular data under low temperature may be attributed to the growth rate–temperature relationship. In contrast, the divergence between the data during the high‐temperature range was likely due to the morphological study (Li et al., 2023) overlooking the small individuals.

Furthermore, the response of foraminifera to temperature appeared to be affected by water depth, as indicated by the data in Figure 3. In particular, the foraminiferal read counts from A5 and C4 stations increased with the temperature. However, the foraminiferal read counts from D6 station plateaued at 24°C and even declined at 30°C. It is important to note that read counts of foraminifera cannot be directly equated with their abundance, but they do reflect relative abundance accurately. The decrease in foraminifera read counts at Station D6 at 30°C indicates a decrease in foraminifera abundance, suggesting a critical point for foraminifera tolerance to high temperatures around 30°C. Notably, this trend was not observed in the foraminifera communities at stations A5 and C4, indicating differences in temperature tolerance. This finding suggested that foraminifera communities at deep water depths have a low tolerance to temperature stress and was consistent with the synchronous morphological data (Li et al., 2023).

### 4.2 Relationship with temperature and its indication

In 1954, Chave first revealed the positive correlation between foraminiferal shell Mg content and sea temperature, which is similar to the results in this study. On the basis of their molecular sequencing data, read counts, OTU counts, and Margalef index of foraminifera were significantly positively correlated with temperature. The relationship between foraminiferal molecular parameters and temperature was first established through regression analysis as Y = a * X + b (Figure 2). Foraminifera changed their growth rate or adopted the r‐strategy during different temperature ranges, resulting in a positive relationship between the read counts of foraminifera and temperature. The OTU counts and Margalef index reflect the diversity of foraminifera. Globally, biodiversity is the highest at low latitudes (Krug et al., 2009), as confirmed by a wide range of taxa, including protists, trees, ants, woodpeckers, primates, and marine benthic animals (Gaston, 1996; Stevens, 1989) found in these locations. This phenomenon suggests a potential relationship between warm climates and high biodiversity. The elevated biodiversity in these regions can be attributed to relatively high temperatures, increased availability of water, and other favorable factors. In this study, the Margalef index of foraminifera increased with the temperature, indicating an improvement in foraminiferal diversity (Figures 2 and 3). The corresponding morphological data in this experiment (Li et al., 2023) also supported the positive correlation between the Margalef index and temperature (Figure 1), which was consistent with a previous morphological study (Dong et al., 2019b). We need to remind future researchers that the foraminifera community used in our experiment was collected from marine environments near 24°C. In the cultivation experiment, the foraminifera community performed better near 24°C, indicating a potentially better adaptation of these communities to the original temperature. Therefore, conducting cultivation experiments on foraminifera communities from different regions and temperatures in the future will further our understanding of foraminifera's response to temperature changes.

The relationship between modern foraminifera and temperature for paleoenvironment reconstruction in the continental shelf has been poorly understood (Antão et al., 2020). Environmental DNA has shown great potential in studying the relationship between foraminifera and the environment and for environmental reconstruction (Frontalini et al., 2018; Pawłowska et al., 2016; Pawlowski et al., 2014). In this study, the molecular adaptation of foraminifera in the continental shelf to temperature change was revealed using eDNA analysis. Results showed foraminifera communities from different water depth stations exhibited different adaptabilities when faced with the same high‐temperature stress (30°C). Foraminifera communities from deep water stations had poor adaptability, and those from shallow water stations showed strong adaptability by adjusting their survival strategies (r strategies) to high‐temperature stress. Furthermore, this study established the quantitative relationship between temperature and foraminifera parameters as follows: Y = 1314.75*X + 44754.51 (read counts); Y = 1.13*X + 44.26 (OTU counts); Y = 1.13*X + 44.26 (Margalef index), which can be used for palaeotemperature reconstruction. Particularly, the read counts of Rotaliida (calcareous foraminifera) exhibited a significantly positive relationship with culture temperature. This finding provides new insights into foraminiferal paleothermometers beyond traditional shell analysis. Compared to traditional approaches, eDNA method is simpler, faster, and more accurate. It eliminates the need for complex sample processing steps for morphological characterization, lengthy species classification and identification, and the handling of small or destructive individuals.

## 5 CONCLUSION

This study aimed to explore the molecular response of benthic foraminiferal communities to temperature across varying water depths through laboratory‐controlled culture experiments. Results revealed a diverse molecular adaptation of foraminifera to different temperature ranges. At low temperatures, foraminifera regulated their growth rate; at high temperatures, they adopted the r‐strategy to cope with temperature stress. This study also found that foraminiferal communities from deep water stations had a low tolerance to temperature stress. This work is the first to introduce the novel concept of utilizing foraminifera's molecular parameters as temperature indicators and establishing the relationship equations: foraminiferal read counts =1314.75*T + 44754.51; OTU counts =1.13*T + 44.26; Margalef index =1.13*T + 44.26. These findings will enhance our understanding of the molecular adaptation mechanism of foraminifera to temperature change and provide new insights into long‐term paleotemperature reconstruction.

## AUTHOR CONTRIBUTIONS

**Haotian Li:** Formal analysis (equal); methodology (equal); software (equal); writing – original draft (equal). **Yanli Lei:** Conceptualization (equal); funding acquisition (equal); supervision (equal); writing – review and editing (equal). **Wenlong Fa:** Conceptualization (equal); data curation (equal); investigation (equal). **Tianzhen Wu:** Data curation (equal); formal analysis (equal). **Tiegang Li:** Funding acquisition (equal); supervision (equal); writing – review and editing (equal).

## CONFLICT OF INTEREST STATEMENT

The authors declare that there is no conflict of interest regarding the publication of this article.

## Supporting information


Data S1.

File S1.

## Data Availability

Data will be made available under accession number PRJNA675069 of the Sequence Read Archive (https://www.ncbi.nlm.nih.gov/sra/PRJNA1068282).
