# Supplementary material for: Environmental DNA sheds new insight on molecular adaptation of foraminifera to temperature from laboratory‐controlled culture experiment
Source: Ecol Evol. 2024 Oct 10;14(10):e70243. doi: 10.1002/ece3.70243 (PMC11464909; doi:10.1002/ece3.70243)
Supplement: Supplementary file 1 — Data S1. [file ECE3-14-e70243-s001.zip › jzhw molecular adaptation--Supplementary data.docx]

**Supplementary data**

**Figure S1** The sampling and laboratory culture information in this study. A: sampling stations; B: sampling tools; C: culture treatments with three replicates at station A5 (water depth of 9.2 m), C4 (water depth of 6.0 m), and D6 (water depth of 26.0 m).

**Table S1** The sequencing data parameters of foraminifera in temperature treatments (6, 12, 18, 24, and 30°C) at different stations (A5: water depth of 9.2 m, C4: water depth of 6.0 m, and D6: water depth of 26.0 m).

| **Station** | **Treatment** | **Replicate** | **Raw Sequences** | **Clean Sequences** | **Effective Sequences** | **Effective%** |
| --- | --- | --- | --- | --- | --- | --- |
| A5 | *in situ* | 1 | 99466 | 85566 | 85566 | 79.18 |
| A5 | *in situ* | 2 | 92292 | 92028 | 92027 | 83.93 |
| A5 | 6°C | 1 | 52693 | 46145 | 46145 | 77.64 |
| A5 | 6°C | 2 | 36906 | 36881 | 36304 | 88.68 |
| A5 | 6°C | 3 | 102058 | 101456 | 96243 | 88.51 |
| A5 | 12°C | 1 | 66677 | 66500 | 66010 | 91.37 |
| A5 | 12°C | 2 | 66229 | 66199 | 66049 | 94.35 |
| A5 | 12°C | 3 | 51811 | 48009 | 48007 | 81.85 |
| A5 | 18°C | 1 | 59602 | 59487 | 59473 | 86.77 |
| A5 | 18°C | 2 | 82332 | 75586 | 75268 | 73.77 |
| A5 | 18°C | 3 | 105080 | 93868 | 88673 | 78.54 |
| A5 | 24°C | 1 | 87602 | 87368 | 87368 | 83.99 |
| A5 | 24°C | 2 | 90351 | 86888 | 86888 | 86.62 |
| A5 | 24°C | 3 | 79907 | 79698 | 79697 | 90.38 |
| A5 | 30°C | 1 | 92376 | 92136 | 92136 | 90.94 |
| A5 | 30°C | 2 | 103115 | 102917 | 102917 | 90.22 |
| A5 | 30°C | 3 | 105090 | 104823 | 104822 | 90.02 |
| C4 | *in situ* | 1 | 96223 | 88279 | 88278 | 76.37 |
| C4 | *in situ* | 2 | 83490 | 83334 | 83334 | 82.84 |
| C4 | *in situ* | 3 | 99285 | 78710 | 78708 | 66.88 |
| C4 | 6°C | 1 | 66301 | 66148 | 65802 | 89.77 |
| C4 | 6°C | 2 | 52663 | 52511 | 52424 | 87.65 |
| C4 | 6°C | 3 | 63954 | 63820 | 63597 | 88.36 |
| C4 | 12°C | 1 | 27298 | 27279 | 27205 | 82.1 |
| C4 | 12°C | 2 | 29442 | 29412 | 29351 | 78.13 |
| C4 | 12°C | 3 | 24397 | 24357 | 24261 | 73.28 |
| C4 | 18°C | 1 | 92460 | 78338 | 78338 | 73.83 |
| C4 | 18°C | 2 | 78263 | 77773 | 77773 | 70.06 |
| C4 | 18°C | 3 | 64004 | 63974 | 63717 | 94.41 |
| C4 | 24°C | 1 | 87808 | 87324 | 87316 | 89.14 |
| C4 | 24°C | 2 | 101831 | 98614 | 98501 | 86.19 |
| C4 | 24°C | 3 | 85736 | 85469 | 83875 | 83.2 |
| C4 | 30°C | 1 | 97016 | 96807 | 96807 | 84.19 |
| C4 | 30°C | 2 | 100322 | 100071 | 100071 | 84.66 |
| C4 | 30°C | 3 | 90592 | 90386 | 90386 | 82.63 |
| D6 | *in situ* | 1 | 99729 | 82166 | 82166 | 69.45 |
| D6 | *in situ* | 2 | 83902 | 68667 | 68667 | 62.38 |
| D6 | *in situ* | 3 | 92229 | 92033 | 92032 | 85.85 |
| D6 | 6°C | 1 | 62704 | 62579 | 62313 | 86.14 |
| D6 | 6°C | 2 | 84420 | 81096 | 81096 | 92.34 |
| D6 | 6°C | 3 | 62964 | 62905 | 62655 | 94.64 |
| D6 | 12°C | 1 | 66549 | 66341 | 66338 | 80.14 |
| D6 | 12°C | 2 | 18784 | 17180 | 18695 | 70.22 |
| D6 | 12°C | 3 | 12332 | 12331 | 12315 | 91.21 |
| D6 | 18°C | 1 | 94144 | 93928 | 93927 | 87.32 |
| D6 | 18°C | 2 | 87914 | 87727 | 87704 | 84.91 |
| D6 | 18°C | 3 | 90131 | 86083 | 85610 | 81.55 |
| D6 | 24°C | 1 | 89715 | 89502 | 89502 | 92.49 |
| D6 | 24°C | 2 | 87234 | 86271 | 86271 | 91.4 |
| D6 | 24°C | 3 | 88326 | 83406 | 83406 | 89.2 |
| D6 | 30°C | 1 | 37503 | 37081 | 37160 | 84.76 |
| D6 | 30°C | 2 | 52795 | 52708 | 52708 | 94.98 |
| D6 | 30°C | 3 | 105888 | 105715 | 105713 | 95.18 |

**Table S2** The foraminiferal parameters in temperature treatments (6, 12, 18, 24, and 30°C) at different stations (A5: water depth of 9.2 m, C4: water depth of 6.0 m, and D6: water depth of 26.0 m).

| **Station** | **Treatment** | **Replicates** | **read counts** | **OTU counts** | **Margalef index** | **Shannon-Wiener index** |
| --- | --- | --- | --- | --- | --- | --- |
| A5 | *in situ* | 1 | 79694 | 1009 | 63.42 | 7.06 |
| A5 | *in situ* | 2 | 85664 | 1101 | 72.27 | 7.67 |
| A5 | 6°C | 1 | 45278 | 229 | 12.68 | 2.70 |
| A5 | 6°C | 2 | 23189 | 354 | 30.18 | 4.70 |
| A5 | 6°C | 3 | 69435 | 1490 | 110.43 | 8.67 |
| A5 | 12°C | 1 | 61198 | 613 | 42.42 | 5.54 |
| A5 | 12°C | 2 | 61710 | 567 | 38.16 | 5.45 |
| A5 | 12°C | 3 | 44896 | 855 | 61.23 | 6.77 |
| A5 | 18°C | 1 | 54470 | 1344 | 82.55 | 7.60 |
| A5 | 18°C | 2 | 65421 | 1565 | 96.00 | 7.82 |
| A5 | 18°C | 3 | 85985 | 391 | 24.82 | 4.24 |
| A5 | 24°C | 1 | 80930 | 1504 | 87.25 | 7.92 |
| A5 | 24°C | 2 | 80771 | 1317 | 75.12 | 7.31 |
| A5 | 24°C | 3 | 73652 | 1387 | 82.55 | 7.67 |
| A5 | 30°C | 1 | 83085 | 1474 | 85.94 | 7.01 |
| A5 | 30°C | 2 | 94332 | 1528 | 84.08 | 7.01 |
| A5 | 30°C | 3 | 96573 | 1422 | 75.12 | 6.62 |
| C4 | *in situ* | 1 | 82529 | 1168 | 71.95 | 7.40 |
| C4 | *in situ* | 2 | 79124 | 1171 | 68.88 | 7.21 |
| C4 | *in situ* | 3 | 74825 | 1038 | 67.24 | 7.23 |
| C4 | 6°C | 1 | 59900 | 561 | 36.74 | 5.15 |
| C4 | 6°C | 2 | 44627 | 478 | 35.32 | 5.12 |
| C4 | 6°C | 3 | 57676 | 544 | 36.19 | 4.97 |
| C4 | 12°C | 1 | 20272 | 531 | 46.36 | 5.06 |
| C4 | 12°C | 2 | 23428 | 418 | 34.33 | 3.83 |
| C4 | 12°C | 3 | 18476 | 413 | 36.85 | 4.38 |
| C4 | 18°C | 1 | 73444 | 1252 | 75.55 | 7.18 |
| C4 | 18°C | 2 | 69189 | 1254 | 76.76 | 7.08 |
| C4 | 18°C | 3 | 59788 | 406 | 26.24 | 2.73 |
| C4 | 24°C | 1 | 73348 | 1300 | 73.37 | 6.22 |
| C4 | 24°C | 2 | 91452 | 1533 | 83.32 | 6.97 |
| C4 | 24°C | 3 | 77534 | 1476 | 90.75 | 8.03 |
| C4 | 30°C | 1 | 89770 | 1527 | 89.55 | 7.63 |
| C4 | 30°C | 2 | 93611 | 1338 | 68.88 | 6.97 |
| C4 | 30°C | 3 | 84398 | 1353 | 76.87 | 6.90 |
| D6 | *in situ* | 1 | 72198 | 1122 | 67.68 | 5.98 |
| D6 | *in situ* | 2 | 61069 | 1053 | 67.13 | 5.30 |
| D6 | *in situ* | 3 | 81528 | 1129 | 66.70 | 6.36 |
| D6 | 6°C | 1 | 55709 | 570 | 40.67 | 5.41 |
| D6 | 6°C | 2 | 77583 | 461 | 29.08 | 4.78 |
| D6 | 6°C | 3 | 56394 | 553 | 38.82 | 5.83 |
| D6 | 12°C | 1 | 59030 | 1238 | 77.85 | 6.80 |
| D6 | 12°C | 2 | 13873 | 390 | 38.60 | 4.58 |
| D6 | 12°C | 3 | 9375 | 346 | 37.72 | 4.14 |
| D6 | 18°C | 1 | 86992 | 1446 | 83.54 | 7.54 |
| D6 | 18°C | 2 | 80108 | 1546 | 91.52 | 7.70 |
| D6 | 18°C | 3 | 79154 | 1480 | 83.97 | 7.46 |
| D6 | 24°C | 1 | 83997 | 1389 | 72.71 | 5.49 |
| D6 | 24°C | 2 | 81553 | 1304 | 72.16 | 5.62 |
| D6 | 24°C | 3 | 79945 | 1161 | 58.61 | 4.15 |
| D6 | 30°C | 1 | 33778 | 251 | 21.54 | 3.86 |
| D6 | 30°C | 2 | 50036 | 924 | 60.57 | 5.68 |
| D6 | 30°C | 3 | 100330 | 1048 | 55.11 | 5.77 |

**Table S3** The read counts of foraminiferal shell groups in temperature treatments (6, 12, 18, 24, and 30°C) at different stations (A5: water depth of 9.2 m, water depth of C4: 6.0 m, and water depth of D6: 26.0 m).

| **Station** | **Treatment** | **Replicates** | **Rotaliida** | **Textulariida** | **Monothalamids** | **Miliolida** |
| --- | --- | --- | --- | --- | --- | --- |
| A5 | *in situ* | 1 | 31281 | 1948 | 42239 | 203 |
| A5 | *in situ* | 2 | 24335 | 1827 | 55030 | 227 |
| A5 | 6°C | 1 | 752 | 115 | 43481 | 2 |
| A5 | 6°C | 2 | 3942 | 88 | 16388 | 15 |
| A5 | 6°C | 3 | 4089 | 199 | 11452 | 11 |
| A5 | 12°C | 1 | 9960 | 879 | 41100 | 101 |
| A5 | 12°C | 2 | 8982 | 766 | 47593 | 65 |
| A5 | 12°C | 3 | 19202 | 3895 | 19408 | 54 |
| A5 | 18°C | 1 | 17928 | 1669 | 32352 | 104 |
| A5 | 18°C | 2 | 19981 | 3744 | 34975 | 101 |
| A5 | 18°C | 3 | 493 | 107 | 1723 | 0 |
| A5 | 24°C | 1 | 26933 | 3173 | 44523 | 81 |
| A5 | 24°C | 2 | 21155 | 3584 | 49307 | 81 |
| A5 | 24°C | 3 | 26841 | 2133 | 38049 | 73 |
| A5 | 30°C | 1 | 32574 | 1666 | 39077 | 653 |
| A5 | 30°C | 2 | 41571 | 2077 | 45391 | 575 |
| A5 | 30°C | 3 | 41700 | 3012 | 44514 | 2068 |
| C4 | *in situ* | 1 | 18027 | 9555 | 48794 | 425 |
| C4 | *in situ* | 2 | 16253 | 20851 | 37704 | 66 |
| C4 | *in situ* | 3 | 24695 | 4846 | 40023 | 72 |
| C4 | 6°C | 1 | 8597 | 271 | 45499 | 23 |
| C4 | 6°C | 2 | 6828 | 262 | 34132 | 22 |
| C4 | 6°C | 3 | 9669 | 326 | 43660 | 42 |
| C4 | 12°C | 1 | 3554 | 135 | 5523 | 6 |
| C4 | 12°C | 2 | 2067 | 58 | 3024 | 2 |
| C4 | 12°C | 3 | 2115 | 282 | 3912 | 10 |
| C4 | 18°C | 1 | 28151 | 10859 | 29411 | 55 |
| C4 | 18°C | 2 | 28726 | 2748 | 32840 | 44 |
| C4 | 18°C | 3 | 35973 | 903 | 17725 | 179 |
| C4 | 24°C | 1 | 39290 | 3496 | 21775 | 126 |
| C4 | 24°C | 2 | 23437 | 7538 | 47546 | 108 |
| C4 | 24°C | 3 | 17125 | 5994 | 33549 | 141 |
| C4 | 30°C | 1 | 31856 | 6016 | 45444 | 227 |
| C4 | 30°C | 2 | 39873 | 6954 | 37003 | 481 |
| C4 | 30°C | 3 | 39761 | 2520 | 34843 | 227 |
| D6 | *in situ* | 1 | 10787 | 1609 | 42092 | 155 |
| D6 | *in situ* | 2 | 5146 | 1459 | 47151 | 150 |
| D6 | *in situ* | 3 | 9240 | 1237 | 61622 | 157 |
| D6 | 6°C | 1 | 12497 | 833 | 38847 | 41 |
| D6 | 6°C | 2 | 19314 | 407 | 53708 | 37 |
| D6 | 6°C | 3 | 14086 | 2233 | 36863 | 243 |
| D6 | 12°C | 1 | 8584 | 509 | 24498 | 110 |
| D6 | 12°C | 2 | 596 | 47 | 4656 | 4 |
| D6 | 12°C | 3 | 821 | 34 | 2092 | 13 |
| D6 | 18°C | 1 | 21346 | 2813 | 57186 | 119 |
| D6 | 18°C | 2 | 20312 | 2461 | 50390 | 102 |
| D6 | 18°C | 3 | 15473 | 2440 | 46322 | 194 |
| D6 | 24°C | 1 | 15895 | 5161 | 20056 | 78 |
| D6 | 24°C | 2 | 16894 | 5133 | 20305 | 78 |
| D6 | 24°C | 3 | 11131 | 3987 | 11502 | 54 |
| D6 | 30°C | 1 | 11653 | 10 | 2617 | 18 |
| D6 | 30°C | 2 | 24548 | 1249 | 9496 | 19 |
| D6 | 30°C | 3 | 60534 | 3405 | 17351 | 27 |

**Figure S2** Rarefaction curve based on the number of OTUs and reads under different temperatures (6, 12, 18, 24, and 30°C) at different stations (A5, C4, and D6). The colorful curves represent different samples. The solid lines were drawn based on the abundance of obtained data, and the dotted lines were drawn based on the abundance of extrapolated data.
